# Impacts of Increasing Additions of Choline Chloride on Growth Performance and Carcass Characteristics of Broiler Chickens Reared to 66 Days of Age

**DOI:** 10.3390/ani12141808

**Published:** 2022-07-14

**Authors:** Caroline R. Gregg, Oscar J. Tejeda, Lindsey F. Spencer, Allan J. Calderon, Dianna V. Bourassa, Jessica D. Starkey, Charles W. Starkey

**Affiliations:** Department of Poultry Science, Auburn University, Auburn, AL 36849, USA; crg0066@auburn.edu (C.R.G.); ojt0001@auburn.edu (O.J.T.); lfs0010@auburn.edu (L.F.S.); ajc0078@auburn.edu (A.J.C.); dvb0006@auburn.edu (D.V.B.); jessica.starkey@auburn.edu (J.D.S.)

**Keywords:** broiler chicken, carcass characteristics, choline chloride, growth performance

## Abstract

**Simple Summary:**

Recommendations for choline intake in broiler chickens are potentially outdated and do not include work investigating the entire grow-out period. Broilers were fed increasing concentrations of additional choline chloride above what was provided by a basal corn- and soybean meal-based diet ingredients, reared for 66 days, and then processed. Supplemental choline had no impact on broiler growth performance or breast myopathy incidence, but it altered carcass part yields. These results suggest that feeding additional choline may not be beneficial for broilers grown to heavier market weights in the absence of environmental and dietary stress.

**Abstract:**

The most recent research cited by the NRC Nutrition Requirements of Poultry to establish choline recommendations was published in 1987, so choline guidelines for modern broilers are outdated and may be insufficient to optimize growth. The objective was to determine the effect of additional dietary choline chloride supplementation on growth performance and carcass characteristics of modern broilers reared for 66 days. As-hatched Ross 708 × Yield Plus broiler chicks (n = 2160; 30 birds per pen) were randomly allotted to one of six experimental corn and soybean meal-based diets formulated to contain an additional 0, 400, 800, 1200, 1600, or 2000 mg of choline chloride above the choline content of the basal diet ingredients. Diets were fed in four phases, and birds were processed at day 66 of age. Growth performance and breast myopathy incidence was not impacted by added choline. While there were differences in breast, wing, thigh, and drum yields, the effects of added choline were not linear. Supplemental choline chloride was not beneficial for growth performance but did impact the carcass characteristics of modern, large frame broilers reared for 66 days.

## 1. Introduction

Choline is a quaternary amine that is involved in multiple important metabolic functions including lipid metabolism, cell signaling, and the generation of methyl groups for biosynthesis. While de novo synthesis of choline is possible, it is not sufficient to meet requirements [1]. This necessitates a dietary source of choline. Soybean meal, a common feedstuff for broiler chickens, is a good source of bioavailable choline [2], so symptoms of choline deficiency in commercial broilers appear to be rare. The NRC Nutrient Requirements of Poultry [3] recommends 1300 mg of choline per kg of feed in broiler diets on an as-fed basis when concentrations of sulfur amino acids are adequate. However, the most recent NRC guidelines were published in 1994 with the most recent experiment cited to determine choline recommendations throughout the entire growth period (d 1 to 56) being published in 1961 [4]. As broiler genetics improve to produce more efficient and larger framed birds, nutrient requirements are likely changing as well. Therefore, current choline recommendations may be insufficient to support optimal growth in modern, high-yielding broiler genetic lines. 

Determining definitive choline recommendations can be challenging as the biosynthesis pathways of choline and the sulfur amino acids, methionine (Met) and cystine (Cys), are connected via the S-adenosyl methionine (SAM-e) cycle [5]. Choline enters the SAM-e cycle upon the irreversible oxidation to betaine. Betaine transfers methyl groups to a derivative of folic acid in order to synthesize Met, which can then be used to regenerate SAM-e [6]. Therefore, choline requirements depend upon the availability of sulfur amino acids, betaine, and folic acid [7]. Feeding betaine in broiler diets has been evaluated as a potential replacement for choline supplementation [8,9,10]. While additional dietary betaine may spare choline’s function in one-carbon metabolism, and partially alleviate heat stress in broilers due to osmoregulatory functions [11], it cannot be converted back to choline [12]. Therefore, betaine cannot substitute for choline’s other metabolic functions.

Current literature on the impacts of supplementing corn- and soybean meal-based diets with additional choline on broiler growth performance has yielded conflicting results. When Met was fed at adequate concentrations, additional dietary choline increased feed intake without altering the feed conversion ratio (FCR) [13] but was also observed to reduce feed intake and improve FCR [14]. De Lima et al. [15] demonstrated that dietary choline below the recommended level dramatically reduces feed intake and gain. They determined the dietary choline requirement for broilers grown to 21 days to be 1046 mg/kg, and above this concentration, improvements in gain appear to diminish [15]. Gregg et al. observed no alterations in the growth performance or carcass traits of straight-run broilers with increasing dietary choline when reared to 32 days in the absence of heat and nutrient stress [16]. Due to a relatively short growth period and light harvest weights in the former experiment, it remains unknown how additional choline will impact modern broilers grown to heavier market weights. However, the most recent NRC proposes that broiler choline requirements diminish with age [3]. Therefore, the objective of this study was to evaluate the impact of increasing additions of choline chloride on the growth performance and carcass characteristics of broilers grown from 0 to 66 d of age.

## 2. Materials and Methods

All procedures regarding live birds were approved by the Auburn University Institutional Animal Care and Use Committee (PRN 2017-3013).

### 2.1. Diet Formulation

An industry standard, antibiotic-free, corn- and soybean meal-based basal diet was formulated to meet the primary breeder’s nutrient recommendations [17]. The basal diet was formulated to provide adequate Met in all phases to isolate the effects of dietary choline. Six experimental diets were then formulated to contain an additional 0, 400, 800, 1200, 1600, and 2000 mg of additional choline chloride per kg of feed. Broilers were fed in 4 dietary phases: starter from day 0 to 15 (crumble), grower 1 from day 16 to 32 (pellet), grower 2 (pellet) from day 29 to 49, and finisher from day 50 to 66 (pellet). Table 1 displays diet composition by feeding phase and Table 2 displays analyzed choline concentrations by dietary treatment by feeding phase.

### 2.2. Broiler Husbandry, Growth Performance, and Carcass Part Yields

As-hatched, Aviagen Ross 708 × Yield Plus broiler chicks were transported from a commercial hatchery to the Auburn University Charles C. Miller Jr. Poultry Research and Education Center and reared to 32 d in a solid-sided, plenum-style house with 30 birds per 2.23 m^2^ pen bedded with new wood shaving litter as previously described by Gregg et al., as the studies were conducted concurrently [16]. Temperature set point was 33 °C on day 0 and was gradually reduced over time based on bird comfort to a final set point of 20 °C. Birds were provided 23 h of light at 30 lux and 1 h of dark from d 0 to 7, 16 h of light at 10 lux and 8 h of dark from d 8 to 15, and 16 h of light at 5 lux and 8 h of dark from d 16 to 66. Feed and water were provided ad libitum. On day 32, birds from half of the pens (n = 2160 birds) were removed for processing. The remaining birds (n = 2160) were then randomly reallotted to new pens within the same dietary treatment to maintain the original number of replicate pens with a reduced stocking density (24 replicates per treatment; 15 birds per pen) for the remainder of the 66-day experiment.

At the end of each dietary phase, individual bird and feeder weights were obtained with mortality recorded on a daily basis. Mortality-corrected BW gain (BWG), feed intake (FI), and feed conversion ratio (FCR) were then calculated. On day 66, birds from 12 replicate pens per treatment (n = 1080; 15 birds per pen per treatment) were fasted for 8 h and processed at the Auburn University Pilot Processing Plant. After a 3 h static, water bath chill, carcass weights without giblets (WOG), and abdominal fat pad weights were recorded. Carcasses were then chilled overnight and deboned by professional deboners the following day. Whole boneless, skinless breast, tender, skin-on, bone-in wings, boneless, skinless thigh, and bone-in drumstick weights were determined.

### 2.3. Wooden Breast and White Striping Scoring

A 4-point scale (0 = normal, 1 = mild, 2 = moderate, and 3 = severe) was used to score breast fillets for the severity of wooden breast (WB) and white striping (WS) as previously described [18]. Hand palpation to determine the proportion of the fillet affected by abnormal hardness was used to evaluate for WB, and visual determination of the proportion of the fillet affected by white striations was used to determine WS severity. Score 0 fillets were free of defects, score 1 fillets were up to ¼ affected, score 2 fillets were up to ½ affected, and score 3 fillets were greater than ½ affected. The same trained and experienced evaluator scored all fillets for both defects. WB and WS severity scores are shown as a proportion of all breasts within dietary treatment groups.

### 2.4. Statistical Analysis

All experimental data were analyzed as a one-way ANOVA using the GLIMMIX procedure of SAS software, ver. 9.4 with pen serving as the experimental unit. Dietary treatment was the fixed effect, and the Satterthwaite adjustment was used to correct degrees of freedom. Proportional data (e.g., mortality and carcass yields) were analyzed using the events/possible events syntax with a binomial distribution and R-side covariance structure in SAS. Means were separated using the PDIFF option. Means were declared different when *p* ≤ 0.05, and tendencies were declared when 0.05 < *p* ≤ 0.10.

## 3. Results

### 3.1. Broiler Growth Performance

Body weight, BW gain, feed intake, and FCR were not impacted by supplemental choline chloride in the diet, as displayed in Table 3. Mortality (Table 4) was also not affected by increasing additions of choline chloride regardless of the growth period.

### 3.2. Carcass Characteristics

Carcass and part weights were not impacted by supplemental dietary choline (Table 5). When set as a proportion of the chilled WOG weight, breast, thigh, and drumstick yields were altered by additional choline treatments in a way that does not appear to suggest a dose-dependent response. The incidence of WB and WS were also similar among treatments.

## 4. Discussion

While no differences were observed in broiler growth performance, it is important to note that the high basal concentrations of choline in the diet may have impacted the ability to determine meaningful differences in growth performance. Additionally, the use of commercially sourced, straight-run chicks likely increased the variation within treatments. This is reflected in the high standard error obtained from these data. This variability may have impacted the ability to detect differences in growth performance parameters. The experimental basal diet utilized in this study met or exceeded the required dietary Met established by the primary breeder [17]. This may explain the observed lack of response to increasing additions of choline chloride due to the connected biosynthesis pathways of Met and choline [19,20,21].

The similar carcass yield across treatments observed in this study is consistent with Igwe et al. [22] who describe an increase in live weight as added dietary choline increases without differences in the dressed carcass yield. This indicates that supplemental choline may not alter the composition of the carcass regardless of changes in growth performance. However, alterations in carcass part yields where the greatest breast, wing, and thigh yields were observed in birds fed an additional 1600 mg of choline per kg of feed suggest a favorable effect of added choline chloride on carcass part yields in birds grown to heavier market weights without impacting the proportion of abdominal fat. This contrasts with a previous study on broilers processed at 32 days of age where there were no differences in carcass part yields but a trend for diminishing abdominal fat with added choline was reported [16]. Therefore, broilers may respond differently to supplemental choline as they age, and the impacts of dietary choline may be dependent on the length of the growth period and the intended market weight. Similarities in WB and WS incidence are consistent with previous research [16]. The development of WB is highly correlated with an increased growth rate and breast yield [23], so no changes in myopathy incidence were expected when growth performance was unaltered by feeding additional choline in the diet. Almost all breast fillets scored in the moderate or severe categories for WB with no normal fillets found.

Broilers in the present study reached live weights above 5 kg with BWG and FCR 10 to 15% above standards set by the primary breeder [24]. Almost all the resulting breast fillets scored either moderate or severe for WB. This indicates that the birds were reared in conditions that were conducive to optimal growth performance with very little environmental stress. Supplementing higher concentrations of dietary choline may be more beneficial for broilers reared under high environmental temperatures. While Kpodo et al. [25] observed no impacts of dietary choline on mitigating the negative impacts of heat stress on broiler growth performance, the combination of supplemental choline and betaine has been found to partially replace dietary Met in heat-stressed broilers [26]. Therefore, future investigation into the impacts of additional choline in Met-deficient diets on broiler growth performance and carcass characteristics when reared under higher environmental temperatures is warranted.

## 5. Conclusions

The rearing conditions in the present study were favorable to produce rapid growth to heavy market weights in large-frame, modern, commercial broilers. In these experimental conditions, feeding diets with high basal choline concentrations, additional choline chloride in the diet did not significantly benefit growth performance or carcass characteristics of broilers grown for 66 days. Supplemental choline may be more advantageous when birds are reared under stressful conditions that better simulate those observed in commercial broiler houses.

## Figures and Tables

**Table 1:** Ingredient and nutrient composition of the basal broiler chicken diets.

Ingredients, % “as Fed” ^1^	Feeding Phase			
	Starter (Day 0 to 15)	Grower 1 (Day 16 to 28)	Grower 2 (Day 29 to 49)	Finisher (Day 50 to 66)
Corn	48.92	55.83	60.96	63.90
SBM	38.28	32.00	26.00	22.70
DDGS	5.40	5.00	6.00	6.00
Soybean oil	3.00	3.00	3.00	3.25
Dicalcium phosphate	1.96	1.70	1.50	1.50
Limestone	1.18	1.20	1.30	1.39
Salt	0.40	0.42	0.42	0.42
L-Lysine hydrochloride	0.15	0.18	0.22	0.25
DL-Methionine	0.33	0.27	0.22	0.22
L-Threonine	0.07	0.10	0.08	0.08
Mineral premix	0.10	0.10	0.10	0.10
Vitamin premix	0.10	0.10	0.10	0.10
Choline chloride premix ^2^	0.10	0.10	0.10	0.10
Calculated Nutrient Content of Basal Diet				
Crude protein, %	25.06	22.34	20.00	18.61
AME_n_, kcal/kg	3049	3111	3159	3197
Digestible Methionine, %	0.72	0.52	0.45	0.44
Digestible Lysine, %	1.45	1.15	1.02	0.95
Analyzed Nutrient Content of Basal Diet				
Choline, mg per kg	1830	1170	1040	1020
Methionine, %	0.65	0.56	0.51	0.49
Betaine, mg per kg	51.35	45.77	56.87	69.77

^1^ SBM = soybean meal, DDGS = corn dried distillers’ grains with solubles, AME_n_ = apparent metabolizable energy. ^2^ Choline premix contained basal diet and added choline chloride.

**Table 2:** Choline concentration (mg per kg) in broiler chicken diets with increasing additions of choline chloride.

Feeding Phase	Dietary Treatment					
	Added Choline Chloride, mg per kg “as Fed”					
	0	400	800	1200	1600	2000
Starter (Day 0 to 15) Diet Choline, mg per kg	1830	2790	-	3260	3740	4460
Grower 1 (Day 16 to 28) Diet Choline, mg per kg	1170	1790	2530	3220	3870	4630
Grower 2 (Day 29 to 49) Diet Choline, mg per kg	1040	1700	2440	1970	3850	4490
Finisher (Day 50 to 66) Diet Choline, mg per kg	1020	1660	2400	3130	3380	4320

**Table 3:** Effects of choline chloride supplementation on broiler growth performance from 0 to 66 d ^1^.

Variable ^2^	Dietary Treatment							
	Added Choline Chloride, mg per kg “as Fed”							
	0	400	800	1200	1600	2000	SEM ^3^	*p*-Value
d 0 BW, g	43	44	43	43	44	44	0.2	0.5128
d 15 BW, g	567	570	566	573	573	565	6	0.4172
d 28 BW, g	2112	2107	2110	2109	2106	2092	23	0.9882
d 49 BW, g	3895	3953	3907	3947	3893	3876	65	0.9375
d 66 BW, g	5061	5066	5077	5155	5062	5055	101	0.9820
d 0 to 15 MC BWG, g	507	506	509	519	520	507	10	0.5042
d 16 to 28 MC BWG, g	1178	1182	1165	1162	1165	1167	20	0.9656
d 29 to 49 MC BWG, g	3203	3368	3295	3315	3253	3244	73	0.6680
d 50 to 66 MC BWG, g	1627	1748	1752	1842	1853	1771	102	0.6709
d 0 to 28 MC BWG, g	1685	1688	1674	1681	1685	1675	27	0.9971
d 0 to 49 MC BWG, g	4245	4371	4345	4359	4311	4270	83	0.8637
d 0 to 66 MC BWG, g	5872	6119	6097	6201	6164	6041	131	0.5581
d 0 to 15 MC FI, g	599	599	595	600	602	594	4	0.6839
d 16 to 28 MC FI, g	1648	1648	1634	1652	1631	1637	29	0.9820
d 29 to 49 MC FI, g	6908	7017	6988	6990	6949	6993	111	0.9865
d 50 to 66 MC FI, g	3688	3685	3688	3691	3672	3606	60	0.9146
d 0 to 28 MC FI, g	2225	2219	2215	2247	2222	2211	31	0.9544
d 0 to 49 MC FI, g	9133	9235	9203	9237	9170	9203	130	0.9926
d 0 to 66 MC FI, g	12,785	12,859	12,893	12,868	12,800	12,838	208	0.9991
d 0 to 15 MC FCR	1.158	1.161	1.156	1.150	1.153	1.154	0.01	0.7642
d 16 to 28 MC FCR	1.400	1.394	1.403	1.424	1.400	1.404	0.01	0.7085
d 29 to 49 MC FCR	2.159	2.088	2.128	2.114	2.142	2.162	0.03	0.5806
d 50 to 66 MC FCR	2.284	2.173	2.173	2.027	2.001	2.197	0.14	0.7185
d 0 to 28 MC FCR	1.321	1.315	1.323	1.338	1.319	1.321	0.01	0.5107
d 0 to 49 MC FCR	2.156	2.118	2.125	2.124	2.131	2.160	0.03	0.9193
d 0 to 66 MC FCR	2.180	2.105	2.124	2.078	2.082	2.135	0.04	0.3911

^1^ Each treatment was represented by 24 replicate pens (30 birds/pen from d 0 to 34 and 15 birds/pen from d 35 to 66). Broilers received diets provided in 4 phases: starter (d 1 to 15), grower 1 (d 16 to 28), grower 2 (d 29 to 49), and finisher (d 50 to 66). ^2^ BW = body weight, MC = mortality corrected, BWG = body weight gain, FI = feed intake, FCR = feed conversion ratio (feed:gain). ^3^ SEM = highest standard error of the LS mean pair-wise comparisons.

**Table 4:** Effect of choline chloride supplementation on broiler mortality from day 0 to 66 ^1^.

Variable	Dietary Treatment							
	Added Choline Chloride, mg per kg “as Fed”							
	0	400	800	1200	1600	2000	SEM ^2^	*p*-Value
d 0 to 15 mortality, %	4.44	7.50	5.28	5.00	5.28	5.42	1.47	0.6512
d 16 to 28 mortality, %	5.29	7.69	4.44	3.49	2.87	6.08	1.90	0.2774
d 29 to 49 mortality, %	1.75	1.16	1.14	1.99	1.41	2.28	0.73	0.7355
d 50 to 66 mortality, %	2.47	1.27	2.48	1.84	3.59	1.90	1.31	0.7431
d 0 to 28 mortality, %	4.72	4.44	2.50	2.22	1.39	2.50	1.17	0.1111
d 0 to 49 mortality, %	9.47	8.05	4.95	6.04	3.76	6.46	1.97	0.1996
d 0 to 66 mortality, %	10.98	8.81	6.48	7.17	6.02	7.61	2.10	0.4247

^1^ Each treatment was represented by 24 replicate pens (30 birds/pen from d 0 to 34 and 15 birds/pen from d 35 to 66). Broilers received diets provided in 4 phases: starter (d 1 to 15), grower 1 (d 16 to 28), grower 2 (d 29 to 49), and finisher (d 50 to 66). ^2^ SEM = highest standard error of the LS mean pair-wise comparisons.

**Table 5:** Effect of choline chloride supplementation on broiler carcass characteristics at 66 days of age ^1^.

Variable ^2^	Dietary Treatment							
	Added Choline Chloride, mg per kg “as Fed”							
	0	400	800	1200	1600	2000	SEM ^3^	*p*-Value
Chilled WOG WT, g	4067	4047	4074	4127	4066	4106	94	0.9918
Fat Pad WT, g	66	62	63	68	67	62	2	0.2732
Breast WT, g	1230	1190	1188	1287	1264	1215	34	0.1923
Tender WT, g	229	216	220	228	223	218	6	0.3995
Wing WT, g	403	401	394	408	427	411	11	0.4044
Thigh WT, g	525	520	491	522	540	523	18	0.4983
Drumstick WT, g	471	453	440	460	471	467	15	0.6088
Chilled WOG, % of live BW	80.57	80.45	80.52	80.65	80.43	81.25	0.30	0.3928
Fat Pad, % of chilled WOG	1.61	1.53	1.56	1.65	1.64	1.50	0.07	0.4426
Breast, % of chilled WOG	30.68 ^a,b,c^	29.39 ^c^	29.17 ^c^	31.23 ^a,b^	31.7 ^a^	29.81 ^b,c^	0.58	0.0094
Tender, % of chilled WOG	5.72	5.34	5.40	5.53	5.60	5.34	0.14	0.3057
Wing, % of chilled WOG	10.06 ^b^	9.914 ^b^	9.662 ^b^	9.893 ^b^	10.71 ^a^	10.09 ^b^	0.20	0.0083
Thigh, % of chilled WOG	13.09 ^a^	12.86 ^a,b^	12.05 ^b^	12.66 ^a,b^	13.53 ^a^	12.84 ^a,b^	0.32	0.0351
Drumstick, % of chilled WOG	11.74 ^x^	11.18 ^x,y^	10.79 ^y^	11.16 ^x,y^	11.79 ^x^	11.45 ^x,y^	0.26	0.0540
Wooden Breast score 0, %	-	-	-	-	-	-	-	-
Wooden Breast score 1, %	-	0.98	-	-	-	0.99	0.59	1.0000
Wooden Breast score 2, %	50.00	50.00	40.57	42.86	50.00	56.44	6.55	0.4972
Wooden Breast score 3, %	50.00	49.02	59.43	57.14	50.00	42.57	6.75	0.4647
White Striping score 0, %	2.27	0.98	0.00	2.04	0.00	0.99	1.25	0.8868
White Striping score 1, %	31.82	29.41	29.25	31.63	21.00	25.74	5.15	0.5809
White Striping score 2, %	51.14	50.00	42.45	39.80	52.00	44.55	5.20	0.3877
White Striping score 3, %	14.77	19.61	28.30	26.53	27.00	28.71	4.80	0.2342
Mean Wooden Breast score	2.44	2.53	2.52	2.60	2.49	2.36	0.09	0.3417
Mean White Striping score	1.92	1.91	1.97	1.96	2.05	1.97	0.10	0.9051

^1^ Total n = 1080 (180 birds per treatment) ^2^ BW = body weight, WOG = without giblets, chilled carcass weight, WT = weight. ^3^ SEM = highest standard error of the LS mean pair-wise comparisons. ^a,b,c^ Means with different superscripts differ *p* ≤ 0.05 and are considered significantly different. ^x,y,z^ Means with different superscripts differ 0.0501 ≤ *p* ≤ 0.10 and are considered tendencies.

## Data Availability

The data presented in this study are available on request from the corresponding author.
